# Five years of the Early-Career Geriatricians Initiative (ECGI): reflecting on the past, shaping the present, and envisioning the future

**DOI:** 10.1007/s41999-025-01236-6

**Published:** 2025-05-23

**Authors:** Daniel Rosselló-Jiménez, Serdar Ozkok, Sofia Duque, Anne Ekdahl, Karolina Piotrowicz, Mirko Petrovic

**Affiliations:** 1 Geriatrics Department, Terrassa University Hospital, Barcelona, Spain; 2 Division of Geriatrics, Department of Internal Medicine, Istanbul University, Istanbul Medical School, Capa, 34390 Istanbul, Turkey; 3 Faculty of Medicine, Preventive Medicine and Public Health Institute, University of Lisbon, Hospital Cuf Descobertas, Lisbon, Portugal; 4 Geriatric Medicine Section, Department of Clinical Sciences, Helsingborg Hospital, Lunds University, Helsingborg, Sweden; 5 Department of Internal Medicine and Gerontology, Jagiellonian University Medical College, Kraków, Poland; 6 Section of Geriatrics, Department of Internal Medicine and Paediatrics, Ghent University, Ghent, Belgium

**Keywords:** Career mobility, Community networks, Education, Medical, Continuing, Geriatrics, Professional development

## Abstract

**Purpose:**

Despite rising global demand, geriatrics is often overlooked by young physicians due to its perceived complexity and limited career development. Launched by the European Geriatric Medicine Society, the Early Career Geriatricians Initiative (ECGI) aims to counter this perception with its rich educational, research, and leadership opportunities.

**Methods:**

This article provides a narrative overview of the ECGI, highlighting its establishment, key milestones, and impact within the EuGMS, while also outlining its structure, major achievements, and future directions.

**Results:**

The ECGI set strategic objectives to integrate early-career geriatricians into EuGMS activities. Key actions included the establishment of working groups, the introduction of a Rotation Program for international observerships, and the organization of dedicated congress events such as the Comprehensive Geriatric Assessment (CGA) Quiz and the Stefania Maggi Award Symposium. ECGI members were also given opportunities to represent the initiative in international conferences. Since gaining official recognition as a Special Interest Group of EuGMS in 2024, the initiative has been steadily evolving, aiming to establish a more structured, sustainable framework that enhances its long-term impact and effectiveness within the geriatric community.

**Conclusion:**

As it marks its fifth anniversary, the ECGI remains committed to empowering the next generation of geriatricians with helping their professional growth and engagement. The early-career geriatricians are encouraged to join ECGI and contribute to shaping the future of geriatric medicine.

## Key summary points

**Aim:**

To outline the origins, development, and impact of the Early-Career Geriatricians Initiative (ECGI) within the European Geriatric Medicine Society (EuGMS), while showcasing the diverse research, educational, and career opportunities it offers to the next generations of geriatricians.

**Findings:**

ECGI was founded in 2019 to provide networking, research, and mentorship opportunities for early-career geriatricians, leading to structured working groups, congress events, and international collaboration programs over the years. Officially recognized as a Special Interest Group of EuGMS since 2024, the initiative continues to evolve with a focus on greater structure and sustainability.

**Message:**

ECGI invites young geriatricians to strengthen collaboration, education, and professional growth within the EuGMS framework.

## The beginnings of the ECGI: setting initial objectives

The European Geriatric Medicine Society (EuGMS) thrives on diversity in career stages, geographic backgrounds, and gender representation, fostering innovation, collaboration, and excellence in geriatric medicine across Europe. The experienced members bring valuable expertise, while early-career professionals ensure continuity and adapt to emerging scientific trends. Balancing these contributions ensures the society’s growth and adaptability. Recognizing the need to involve more early-career geriatricians, the EuGMS Executive Board discussed strategies to address this. A previous residents’ group had dissolved as members advanced in their careers. To overcome this, the Board led by then-President Prof. Finbarr Martin, enthusiastically supported reactivating the group. To ensure continuity, a senior Board member was appointed as an anchor.

In 2019, the Early-Career Geriatricians Initiative (ECGI) was launched by EuGMS General Secretary at the time, Prof. Anne Ekdahl, and then-President, Prof. Finbarr Martin, along with at that time EAMA Director Prof. Nele Van Den Noortgate and Communications and Website Director Dr. Sofia Duque, highlighting the critical role of communication in engaging early-career geriatricians. The feedback from early-career geriatricians and Full Board recommendations highlighted the need to involve national society members and support clinical and research-focused early-career professionals.

During the 2019 Krakow Executive Board meeting, the group established the following key objectives:

- Support early-career geriatricians in clinical practice,
- Foster research,
- Provide senior mentorship opportunities,
- Increase EuGMS Congress participation,
- Create a dedicated meeting space at congresses.

This Full Board meeting in Krakow in 2019 supported the initiative by tasking members with identifying early-career geriatricians from various countries, followed by a call for involvement through the EuGMS Secretariat.

The group’s first online meeting was held on February 26th, 2020, leading to the formation of working groups like the Blog, V(ideo)-log group, and Reporters groups, which shared updates from partner societies and congress activities.

In its first year, ECGI’s participated in the 2020 EuGMS E-Congress, organizing:

1. A symposium on young geriatricians’ experiences during the COVID-19 pandemic, featuring representatives from Belgium and Spain, followed by a brief report [1],
2. A meeting to discuss future plans and activities within EuGMS.

## Consolidation of the ECGI

In 2021, new working groups emerged, including one focused on mapping early-career activities and national groups across Europe. A major milestone was the introduction of an abstract category at the EuGMS Congress for early-career geriatricians, with top submissions recognized during the Stefania Maggi Award Symposium. The group also held its first in-person meeting in Athens, fostering enthusiasm among participants.

In 2022, the London Congress saw the launch of the Rotation Program, offering two-week exchanges between geriatric departments, and the Comprehensive Geriatric Assessment (CGA) Quiz, now a popular congress event centred on CGA principles.

Since 2023, ECGI has hosted a dedicated symposium within the Congress’s Core Curriculum. From its inception, ECGI has been pivotal in creating and curating content for EuGMS’ official social media channels, further supporting early-career geriatricians (Table 1).

**Table 1:** Working groups within the ECGI and their aims

Working group	Year of creation	Aims
Blog	2020	To write and review short texts for the ECGI blog to present news about geriatrics in the world
EuGMS reporters	2021	To report about interesting topics from different congresses. It allows members to attend other scientific congresses (those societies with which the EuGMS has an active Memorandum of Understanding). This provides opportunities for connections with younger colleagues from these societies for younger congress-participants from across Europe to meet and get to know one another
ECGI papers	2021	To prepare papers and other type of communications (posters, oral communications) related to geriatric medicine, with the management and leadership of ECGI members interested in research
Videologs (vlogs)	2021	To produce video materials concerning EuGMS activities to be shared through EuGMS social media
Mapping	2021	To map activities involving early-career geriatricians across Europe
Rotation program	2022	To provide a framework for early-career geriatricians to have hands-on interactions with other countries and share experiences, skills, and knowledge about geriatric medicine practices across the Europe
CGA Quiz	2022	To create a Quiz which takes place at the EuGMS Conference, where participants are organized by teams and compete answering questions based on CGA

*CGA* comprehensive geriatric assessment, *ECGI* Early Career Geriatricians Initiative, *EuGMS* European Geriatric Medicine Society

## Update on the ECGI progress and developments

**Fig. 1:**
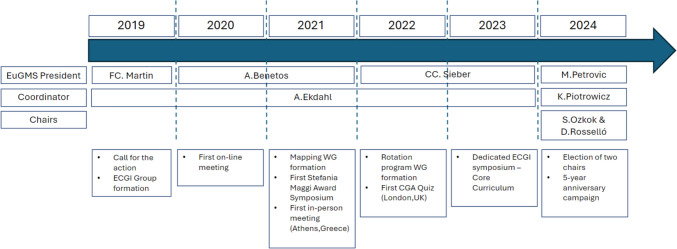
Timeline of the ECGI and its activities. *CGA* comprehensive geriatric assessment, *ECGI* Early Career Geriatricians Initiative, *EuGMS* European Geriatric Medicine Society, *WG* working group

In January 2024, the anchor role of the ECGI transitioned to newly elected General Secretary, Prof. Karolina Piotrowicz. Following discussions on the group regulation and continuity, the Executive Board incorporated the ECGI into the Special Interest Groups (SIG) category in September 2024, aligning it with the EuGMS SIG rules. A formal call for new ECGI Chairs led to the election of Dr. Daniel Rosselló-Jiménez (Spain) and Dr. Serdar Ozkok (Türkiye), who were officially introduced at the EuGMS Congress 2024 in Valencia (Fig. 1).

To enhance communication, the liaison roles were assigned: Prof. Karolina Piotrowicz for the Executive Board, and Prof. Gulistan Bahat for the Academic Board. Over the past year, ECGI continued its impactful initiatives, co-organizing key congress events in Valencia, including the CGA Quiz, a pros and cons Core Curriculum session, the Rotation Program presentation, and the Stefania Maggi Award session. The Vlog Working Group also provided event coverage, maintaining ECGI’s active presence.

## Shaping the future of geriatrics with the ECGI: overcoming barriers, enhancing visibility, and repositioning the specialty for the next generation

The future of the ECGI will undoubtedly be defined by the sociodemographic situation, marked by an increase in the older adult population. This fact will lead to a higher demand for geriatricians from healthcare systems, presenting a challenge in terms of talent retention, particularly for the new generations of geriatric medicine specialists. Shortage of skilled workers and its impact on geriatric medicine is a fact, and the time to raise awareness of possibilities for attracting and retaining healthcare professionals to cope with the expected future increase in demand for geriatric care has definitely arrived [2]. In the United States, for example, geriatrics continues to rank among the least selected specialties by new physicians; in 2025, only 204 of the 382 available fellowship positions in geriatric medicine were filled, leaving nearly half unoccupied [3]. Likewise, a recent Danish survey study reported that only one in eight early-career clinicians who had already completed a year of training in Internal Medicine and Geriatrics reported considering a career in geriatric medicine [4].

The key deterrents contributing to the low preference for geriatrics among young physicians include the perception of lower financial compensation compared to other specialties, the absence of a standardized, homogenous educational curriculum that adequately imparts the knowledge, skills, and professional attitudes required to manage complex geriatric cases, and the field’s perceived low prestige, limited academic advancement, and constrained professional development opportunities [5–7]. While direct salary increases are not yet widespread, countries like the United States and Australia are implementing financial incentives and policy reforms aimed at supporting physicians dealing with older adults—steps that may ultimately enhance the appeal of geriatrics [8, 9].

Initiatives such as PROGRAMMING CA21122 play a pivotal role in supporting the development of standardized and contemporary geriatric medicine curricula in countries where the specialty is still emerging. By offering a pragmatic set of educational and collaborative opportunities, this action aims to strengthen geriatric education and raise awareness among young physicians, policymakers, and the general public—ultimately helping to challenge and transform persistent negative perceptions of geriatrics across multiple levels [10]. The campaigns like #ChooseGeriatrics, which is launched by the British Geriatrics Society (BGS) encourage more healthcare professionals to consider this specialty by showcasing the fulfillment of improving older adults’ health and quality of life and the diverse career opportunities geriatrics offers [11].

Finally, platforms like the ECGI can help counteract the perception of geriatrics as a low-tech or less dynamic discipline by offering access to educational and research opportunities, mentorship, and inspiring role models—and by aligning geriatrics with innovation, leadership, and public health impact. These initiatives will help repositioning the specialty as a prestigious and forward-looking career path capable of addressing the growing global need.

The ECGI aims to build upon its successes while adapting to new generations of geriatricians and exploring new initiatives in order to add significative value to them, such as:

- *Rotation programs*: observerships across offer early-career geriatricians hands-on experience and promote cross-border collaboration, enriching both clinical knowledge and research activity.
- *Dedicated ECGI Symposia at EuGMS congresses:* integrated into the Core Curriculum, these sessions give early-career geriatricians international exposure and career advancement opportunities.
- *Active involvement in esteemed international congresses: r*epresenting ECGI at international scientific conferences encourages shared learning and professional connections.
- *Research and mentorship programs:* ECGI supports member-led international research projects and mentorship programs that pair early-career geriatricians with senior experts, aiding skill development, knowledge sharing, and network building. By prioritizing these initiatives, ECGI can continue to provide valuable career-boosting opportunities and foster growth within the field.
- *Addressing continuing educational needs:* lifelong education is vital in geriatrics and ECGI can focus on creating practical, goal-oriented training materials tailored to the challenges faced by early-career geriatricians.
- *Enhancing visibility through communication:* as an integral part of EuGMS, ECGI should implement a strategic communication plan to increase its prominence and attract more members. Leveraging social media will expand outreach, foster engagement, and strengthen its network. Celebrating ECGI’s fifth anniversary with a dedicated logo can further enhance its identity and draw attention to its achievements.

At a European level, EuGMS is not the one to formally give voice to young professionals with an official group. Other international scientific societies, such as European Federation of Internal Medicine (EFIM) that with Young Internists group has the mission of strengthen the specialty of Internal Medicine by creating a network of young internists, promoting internal medicine, participating in administrative processes, and fostering education and research [12]. Moreover, European Junior Doctors (EJD) is an organization with more than 50 years of history with healthcare digitalisation, postgraduate training, workforce planning, and European cooperation as key pillars [13]. EJD sends representatives to the European Union of Medical Specialists (UEMS) Boards, Sections, and Multidisciplinary Joint Committees, recognizing the vital importance of incorporating the perspectives of doctors in training into the development and evaluation of postgraduate medical education across both institutions [14]. These initiatives show success in attracting young physicians to their respective communities and contributing to improvements in medical education and health systems through engagement, advocacy, and cross-border collaboration. The ECGI aims to serve this unmet need within the field of geriatrics and is well-positioned to generate a comparable impact across Europe.

## Conclusion

ECGI is a vital platform supporting early-career geriatricians through skill development, research opportunities, and networking. To remain dynamic and impactful, it must expand its reach across Europe, enriching its community with diverse perspectives.

The authors invite early-career geriatricians within 5 years of completing their specialty training to join ECGI, gain invaluable experiences, and build a promising career in geriatrics. Colleagues interested in joining are encouraged to contact us via this address for further details and membership inquiries: https://www.eugms.org/about-us/early-career-geriatricians-initiative.html.

## Data Availability

Data sharing not applicable to this article as no datasets were generated or analyzed.
